# Economic opportunity, health behaviours, and health outcomes in the USA: a population-based cross-sectional study

**DOI:** 10.1016/S2468-2667(16)30005-6

**Published:** 2016-10-03

**Authors:** Atheendar S Venkataramani, Rachel Brigell, Rourke O’Brien, Paula Chatterjee, Ichiro Kawachi, Alexander C Tsai

**Affiliations:** Division of General Internal Medicine (A S Venkataramani MD) and Chester M Pierce, MD Division of Global Psychiatry (A C Tsai MD), Massachusetts General Hospital, Boston, MA, USA; Harvard T H Chan School of Public Health, Boston, MA, USA (R Brigell MPH, Prof I Kawachi PhD); La Follette School of Public Affairs, University of Wisconsin-Madison, Madison, WI, USA (R O’Brien PhD); and Department of Medicine, Brigham and Women’s Hospital, Boston, MA, USA (P Chatterjee MD)

## Abstract

**Background:**

Inequality of opportunity, defined as differences in the prospects for upward social mobility, might have important consequences for health. Diminished opportunity can lower the motivation to invest in future health by reducing economic returns to health investments and undermining hope. We estimated the association between county-level economic opportunity and individual-level health in young adults in the general US population.

**Methods:**

In this population-based cross-sectional study, we used individual-level data from the 2009–12 United States Behavioral Risk Factor Surveillance Surveys. Our primary outcomes were current self-reported overall health and the number of days of poor physical and mental health in the last month. Economic opportunity was measured by the county-averaged national income rank attained by individuals born to families in the lowest income quartile. We restricted our sample to adults aged 25–35 years old to match the data used to assign exposure. Multivariable ordinary least squares and probit models were used to estimate the association between the outcomes and economic opportunity. We adjusted for a range of demographic and socioeconomic characteristics, including age, sex, race, education, income, access to health care, area income inequality, segregation, and social capital.

**Findings:**

We assessed nearly 147 000 individuals between the ages of 25 years and 35 years surveyed from 2009 to 2012. In models adjusting for individual-level demographics and county-level socioeconomic characteristics, increases in county-level economic opportunity were associated with greater self-reported overall health. An interdecile increase in economic opportunity was associated with 0·76 fewer days of poor mental health (95% CI −1·26 to −0·25) and 0·53 fewer days of poor physical health (−0·96 to −0·09) in the last month. The results were robust to sensitivity analyses.

**Interpretation:**

Economic opportunity is independently associated with self-reported health and health behaviours. Policies seeking to expand economic opportunities might have important spillover effects on health.

**Funding:**

Robert Wood Johnson Foundation Health and Society Scholars Program.

## Introduction

Inequality of economic opportunity, defined as disparities in the prospects for upward social mobility, has come to the forefront of public discourse in the USA and Europe.^1,2^ Research has shown that for many people in the USA, their life chances are contingent on geographic and temporal factors.^3,4^

In addition to threatening economic status and social values, inequality of opportunity might also adversely affect health in three ways. Economic theories of human capital suggest that reduced economic opportunity can harm health by lowering economic and social returns to health investments.^5^ Restrictions on future possibilities for economic gain make it less probable that the economic benefits of being healthier (eg, higher wages) will actually materialise. An alternate mechanism, informed by the psychology literature, is that reduced economic opportunity diminishes hope, which in turn can undermine health and health behaviours.^6^ Economic opportunity might lead to improved education, employment, income, and access to benefits such as health insurance, all of which have been established as important social determinants of health.^7^ Importantly, these mechanisms can occur independently of any effects of area-level income or income inequality.^4^

The public health relevance of economic opportunity was outlined in a US national study^8^ of county-level data showing robust, positive associations between economic opportunity, health behaviours, and health. However, this study examined aggregate outcomes only, raising concerns for bias resulting from the ecological fallacy. Studies focusing on small samples of Asian Americans and Hispanics have also shown positive associations between perceptions of economic opportunity and health.^9,10^ Nevertheless, whether these findings apply more broadly is unclear. Therefore, in this study, we estimated the association between county-level economic opportunity and individual-level health in adults in the general US population. We focused primarily on measures of self-reported overall, physical, and mental health and on key health behaviours.

## Methods

### Data and study design

Measures of economic opportunity have typically focused on quantification of income mobility across two generations over large geographic areas. Chetty and colleagues^3,11^ created the first county-level estimates of economic opportunity with 2010–12 income tax return data for over 10 million individuals from the 1980–82 birth cohorts to match to similar data for their parents (averaged over 1996–2000, when these 10 million individuals were adolescents). Counties were assigned on the basis of the child’s zip code of residence at the age of 15 years. We used Chetty and colleagues’ measure of absolute upward mobility as our primary exposure variable. This measure denotes, for each county, the average income rank that individuals born to the poorest quartile of parents were able to attain Higher average income ranks reflect greater economic opportunity.

In sensitivity analyses, we also considered two additional measures of economic opportunity. First, we used the county-level intergenerational correlation in income or income rank, also obtained from Chetty and colleagues. This variable reflects the coefficient obtained from fitting a regression model with child income rank as the dependent variable and parental income rank as the exposure. Higher values imply persistence of income ranks—ie, that children of poorer parents are more likely to stay poor whereas children of richer parents are more likely to stay rich. Second, we used Chetty and Hendren’s measure of the expected change in adult income attributable to growing up in a particular county.^12^ This measure, computed with use of the same income tax database, compared differences in adult incomes across siblings in families who moved from one county to another during their childhood. All of these measures are publicly available online through the Equality of Opportunity Database.^13^

We used data from the United States Behavioral Risk Factor Surveillance Survey (BRFSS), a nationally representative random digit telephone-based survey of non-institutionalised adults.^14^ To most closely match the cohorts used by Chetty and colleagues to create the economic opportunity measures, we restricted our sample to individuals aged 25–35 years in the 2009–12 BRFSS. In addition to having a similar age profile as the data in Chetty and colleagues’ study, this choice allowed us to focus on those individuals for whom future prospects for upward mobility are probably most salient—ie, those with the bulk of their economic lives ahead of them.

Overall response rates for the BRFSS ranged from 47–50% over the period 2009–12, which compares favourably to other US national surveys.^15^ Other work has shown that sampling weight-based adjustments for non-response produces representative estimates.^16^

### Outcomes

Our primary outcome of interest was self-reported health. We specifically focused on three measures. The first was the response to the question “Would you say that your general health is excellent, very good, good, fair, or poor?”, captured by a 5 point Likert scale. The remaining two outcomes were the number of days in the last month in which the respondent’s mental health and (separately) physical health were reported as “not good”. These measures were fielded as part of the BRFSS healthy days core, a validated set of questions measuring health-related quality of life.^17^

Our secondary outcomes focused on health behaviours and risk factors. These included a binary indicator of ever smoking, body-mass index (BMI; in kg/m^2^, calculated from self-reported weight and height), and engagement in HIV-related risk behaviours (elicited as a response to a single question asking whether the respondent engaged in any one of the following in the last year: intravenous drug use, treatment for a sexually transmitted infection, receipt of money or drugs in exchange for sex, or unprotected anal intercourse).

### Covariates

We adjusted for several individual and county-level characteristics in our analyses, each motivated by previous research on the social determinants of health.^18–20^ Individual-level demographic variables included age, sex, race (binary variables for black, Hispanic, or other ethnic origin, with white as the reference group), and marital status. We also considered individual socioeconomic characteristics, specifically household income category, and binary indicators for high school and college completion, employment, and health insurance status.

For county-level socioeconomic covariates, we included 2010 income per head, 2010 unemployment rate, and the Gini coefficient measured in 2012. These data were obtained from the US Bureau of Economic Analysis and the US Census Bureau, respectively.^21,22^ We also included the county’s urban–rural classification (counties in metropolitan areas, counties outside of metropolitan areas with an urban population >20 000, counties outside of metropolitan areas with an urban population between 2500 and 20 000, and rural counties, ie, populations <2500), demographic composition (the percentage of the population that was African American, the percentage of the population older than 65 years and 15 years or younger), and population density. These data were drawn from county-level Area Resource File data, which are available at the Inter-University Consortium for Political and Social Research databases.^23^

To account for differences in social structure and marginalisation, we included county-level measures of the violent crime rate per 100 000 for the year 2000 (drawn from Federal Bureau of Investigation Uniform Crime Statistics data, which are available at the Inter-University Consortium for Political and Social Research)^24^ and Rupasingha and Goetz’s social capital index (normalised to zero) that accounts for voter turnout and participation in community organisations over the period 1990–2005.^25^ We also included US census-based measures of residential income segregation and racial segregation, which were obtained from the Equality of Opportunity Project database.^13^ Finally, we included a measure of physicians per head in 2007 from the US Community Health Status Indicators dataset.^26^ Precise descriptions and data sources for all outcome, exposure, and covariate measures are provided in the appendix.

### Statistical analysis

We first estimated unadjusted associations between the outcomes and economic opportunity using descriptive county maps and bivariate regression models. We next fitted multivariable regression models of the following form:

$$Y_{ijs}=g(\alpha_{0}+\alpha_{1}\times {\text{Economic opportunity}}_{js}+\beta X_{ijs}+\gamma Z_{js}+\delta_{s}+\varepsilon_{ijs})$$

Where *i* indexes individuals, *j* indexes counties, and *s* indexes the state of residence. In 2010, the average US county had a population of 100 000 residents, whereas the average US state (which is comprised of counties) has a population of 6·2 million people.^27^ *Y_ijs_* refers to the outcome variable and *g* is either an ordinary least squares or probit link function, depending on whether the outcome is continuous or binary. *Economic opportunity_js_* represents the county-level absolute upward mobility measure. The vectors *X_ijs_* and *Z_ijs_* are comprised of the individual-level and county-level covariates described previously. The term δ_*s*_ denotes state fixed effects, which we included to adjust for macrolevel socioeconomic and institutional factors that might jointly be correlated with economic opportunity and health. All models include survey year and month fixed effects (which are individual specific and denoted in *X_ijs_*). We clustered all standard errors at the level of the county, given that this is the level of variation for our exposure of interest.

Covariate adjustment in this type of research design is prone to both omitted variable bias and overadjustment.^28^ These biases are most likely to materialise with the individual-level socioeconomic characteristics—namely education, household income, employment, and health insurance. These variables might serve as important confounders, but since they might also lie on the causal chain linking economic opportunity and health,^14,29^ including them in the regression model would amount to overadjustment Consequently, we estimated two sets of adjusted models. In the first, we included a minimum set of individual-level (*X_ijs_*) covariates: age, race, sex, marital status, survey month, and year fixed effects. In the second, we added individual-level household income, high school and college completion, employment, and health insurance status. We then assessed the stability of the coefficient estimates across these specifications, reasoning that stable coefficient estimates would suggest the robustness of the results to both omitted variable bias and overadjustment.

We did six sensitivity analyses. First, we examined the consequences of multiple hypothesis testing by implementing a Bonferroni-type p value adjustment that accounts for correlation across each of the dependent variables.^30^ We also assessed the statistical significance of our results using a single index as an outcome, which we defined as the first principal component extracted from a principal components analysis of all of the outcome measures.^31^ Second, we replaced the absolute mobility measure with the alternate measures of opportunity described above (ie, inter-generational correlation in income or income rank and the expected change in adult income attributable to growing up in a particular county). Third, we addressed potential bias from sampling methods by estimating our models separately for the 2011 and 2012 surveys, which were the first BRFSS waves to sample mobile telephone users. Fourth, we addressed the possibility of non-random migration within the past year induced by county economic opportunity using data from the publicly available Current Population Survey and American Community Surveys (appendix). Fifth, given the known geographic concentration of low opportunity in southern states of the USA,^3^ we assessed whether our results were driven by this region by allowing for an interaction between our opportunity measure and a binary indicator of living in the southern US census region. Sixth, we estimated placebo models focusing on individuals past retirement age (aged 65–75 years). We hypothesised that these individuals would be less sensitive to economic opportunity as the bulk of their economic lives are behind them;^8^ thus, null estimates from these models would further underscore the robustness of our study findings.

Prevalence estimates and regression models were weighted to account for the complex sampling design of the BRFSS. All analyses were done using Stata software, version 14. As this study relied solely on data in the public domain, no ethical approval was sought for the study procedures.

### Role of the funding source

The funders of the study had no role in study design, data collection, data analysis, data interpretation, or writing of the report. ASV and ACT had full access to all the data in the study and had final responsibility for the decision to submit for publication.

## Results

Our final sample consisted of nearly 147 000 individuals between the ages of 25 years and 35 years surveyed from 2009 to 2012 and for whom complete case data were available (table 1). The final sample accounts for 78% of all BRFSS observations; an analysis of missing data, which are inconsequential for the results, is shown in the appendix (pp 5, 6). The mean age was 30·5 years, with 50% of respondents being female. Under-represented minorities (black and Hispanic individuals) collectively formed 24% of the sample. 35% of individuals reported completing college and 10% of respondents reported being in fair or poor health. Respondents reported a mean of 2·5 days (SD 6·3) of poor physical health and 4·0 days (SD 7·9) of poor mental health over the past month. 41% of individuals in the sample reported ever smoking and 6% reported having engaged in HIV-transmission risk behaviours in the past year. The mean BMI was 27·34 (SD 5·73).

Our sample was spread across 2242 counties, accounting for over 95% of the total US population in 2010. The mean of absolute mobility, our core measure of economic opportunity, was 41·2 (SD 3·89; range 30·9–63·5). Counties in the top decile of economic opportunity were typically situated in the midwest USA whereas the counties in the lowest decile were situated in the southeast USA, southwest USA, and upper midwest USA (figure). These areas in the lowest decile also had worse self-reported health outcomes.

Higher economic opportunity was associated with improved self-reported health (table 2). In the unadjusted model estimates, an interdecile increase in economic opportunity (eg, equivalent to a shift in residence from southern USA to midwest USA) was associated with a 0·1 point increase in overall self-reported health (b=0·007, 95% CI −0·011 to −0·003), or a 4% relative increase compared with the sample mean. The same interdecile increase in opportunity was associated with 0·71 fewer days of poor mental health (b=−0·049, −0·067 to −0·031) and 0·22 fewer days of poor physical health (b=−0·015, −0·030 to 0·006).

We estimated larger, statistically significant associations for all self-reported health outcomes after adjusting for individual demographics, county characteristics, and state fixed effects (table 2). An interdecile increase in opportunity was associated with a 0·12 point increase in self-reported health (b=0·0085, 95% CI 0·003–0·014), and was associated with decreases in poor physical health of 0·53 days (b=−0·037, 95% CI −0·069 to −0·006) over the past month and poor mental health of 0·76 days (b=−0·0053, −0·088 to −0·018), reported over the past month. In relative terms, the interdecile changes for physical and mental health days represent 20% of the sample means. The estimates for days of poor mental health remained substantively large and statistically significant. Coefficient estimates for covariates are provided in the appendix (pp 7–10). The estimates for days of poor mental health remained substantively large and statistically significant (appendix).

Higher economic opportunity was associated with a reduced likelihood of ever smoking and engaging in HIV-transmission risk behaviours, and lower BMI (table 3). In models adjusting for individual demographics, county characteristics, and state fixed effects, we found that an interdecile increase in opportunity was associated with a 5·7% point decrease in the probability of ever smoking (b=−0·0044, 95% CI −0·0067 to −0·002) and a 2·5% point decrease in the probability of engaging in HIV-transmission risk behaviours (b=−0·0017, −0·0029 to −0·0006). In relative terms, these associations amount to 14% and 40% of the sample mean for each set of behaviours, respectively. The magnitudes of these associations were attenuated with the inclusion of individual socioeconomic characteristics in the regression models (table 3). The negative association with BMI was small in magnitude and not statistically significant in any of the adjusted specifications.

The sensitivity analyses confirmed the robustness of our findings. After the Bonferroni correction, the association between economic opportunity and all outcomes remained statistically significant with the exception of physical health days (appendix p 11). The association between economic opportunity and the first principal component of all outcome variables was negative and statistically significant (appendix p 12). Findings were qualitatively unchanged when refitting the regression models with the alternate measures of opportunity described above (appendix). Our findings remained qualitatively unchanged when we restricted our sample to the 2011–12 data (appendix p 13). We did not find any association between economic opportunity and the probability of having migrated across counties within the past year or across states any time since birth. We also did not find any evidence that healthier individuals were more likely to have migrated into higher opportunity counties over the same timeframe (appendix p 14). We found no evidence of differential regional effects (appendix p 15). Finally, estimates for individuals aged 65–75 years generally showed smaller associations that were not statistically significant (appendix p 16).

## Discussion

In this study, we found that county-level economic opportunity was positively associated with self-reported overall, physical, and mental health in adults aged 25–35 years in the USA. We also found strong inverse associations between economic opportunity and smoking and HIV risk related behaviours. No association was observed between economic opportunity and BMI, which could be because of the complex social patterning of bodyweight in the USA.^32,33^ These associations persisted even after adjustment for multiple individual-level and county-level characteristics and state or year fixed effects. The estimated associations were not only statistically significant but also large in magnitude: the adjusted difference in overall health between the lowest and highest performing counties in terms of opportunity (southeast USA *vs* midwest USA) was equivalent to 18% of the difference in overall health between respondents completing college versus those who did not. Similarly, the estimated interdecile changes in physical and mental health were equivalent to 20% and 34% of the corresponding associations between these variables and individual college education, respectively.

These findings support previous work on the association between economic opportunity and mortality in the USA with the use of county-level data.^8^ The findings also add to a growing evidence base on the social determinants of health, which has thus far primarily focused on the association between area-level income inequality and health.^18,20,34^ Although income inequality might itself be a driver of economic opportunity,^3^ our findings suggest that the association between economic opportunity and health is independent of and distinct from the association between area-level income inequality and health.

Our findings, particularly those related to mental health, might be of relevance to researchers investigating the causes of the rise in mortality in 45–64-year-old whites in the USA.^35^ Some researchers have hypothesised that this alarming increase in mortality is being driven by Americans’ growing despair about the realisation that they might not be better off than their parents, a concept intimately tied to economic opportunity and the “American Dream”.^35^ Additionally, our results might also be of relevance outside of the USA. In particular, several European countries, most notably Italy and the UK^36,37^ have similar levels of overall social mobility as the USA, and the health consequences of future expectations generated by these remain unexplored.

This study has several limitations. First, although we adjusted for a large set of confounders, our core findings might have been affected by omitted variables and reverse causality. Second, non-random migration of healthier individuals to high opportunity areas might have biased our estimates. Although we did not find any correlation between opportunity and migration across counties within the past year or migration across states since the time of birth, bias could still result from within-state, cross-county migration at an earlier stage of life. Third, our measure of economic opportunity was retrospective, in that it reflects already-realised outcomes, and is measured at an aggregate level.^3,4^ The climate of economic opportunity at the time of the survey might have differed from that measured in the data in the study by Chetty and colleagues in 2014. Economic opportunity might also have more salient effects on health at a more local level (such as the census tract or neighbourhood) than at a county level. Fourth, opportunity structures might either be correlated across geographic units (spatial autocorrelation) or affect health outcomes in neighbouring units (spatial lag). Our method of clustering at the county level does not necessarily fully address either possibility, and future work with finer geographic units might consider a spatial regression approach.^38^ Finally, all of our health measures were self-reported and therefore prone to reporting biases.

Each of these limitations motivates avenues for further work. First, future work should examine whether the observed association is causal, perhaps with the use of exogenous variation from policies that ostensibly raise or restrict economic opportunity. Second, studies should use more granular data for individual hopes and aspirations, which will enable an understanding of the underlying behavioural mechanisms and any effect modification by race, ethnicity, or sex. Finally, recent work suggests that the association between economic opportunity and health over the course of the lifecycle might be bidirectional.^3,4,39^ Further elucidating these complex, reinforcing links may provide important insights into how disparities in health and welfare evolve over time and across generations.

## Supplementary Material

Supplemental Appendix

## Figures and Tables

**Figure:**
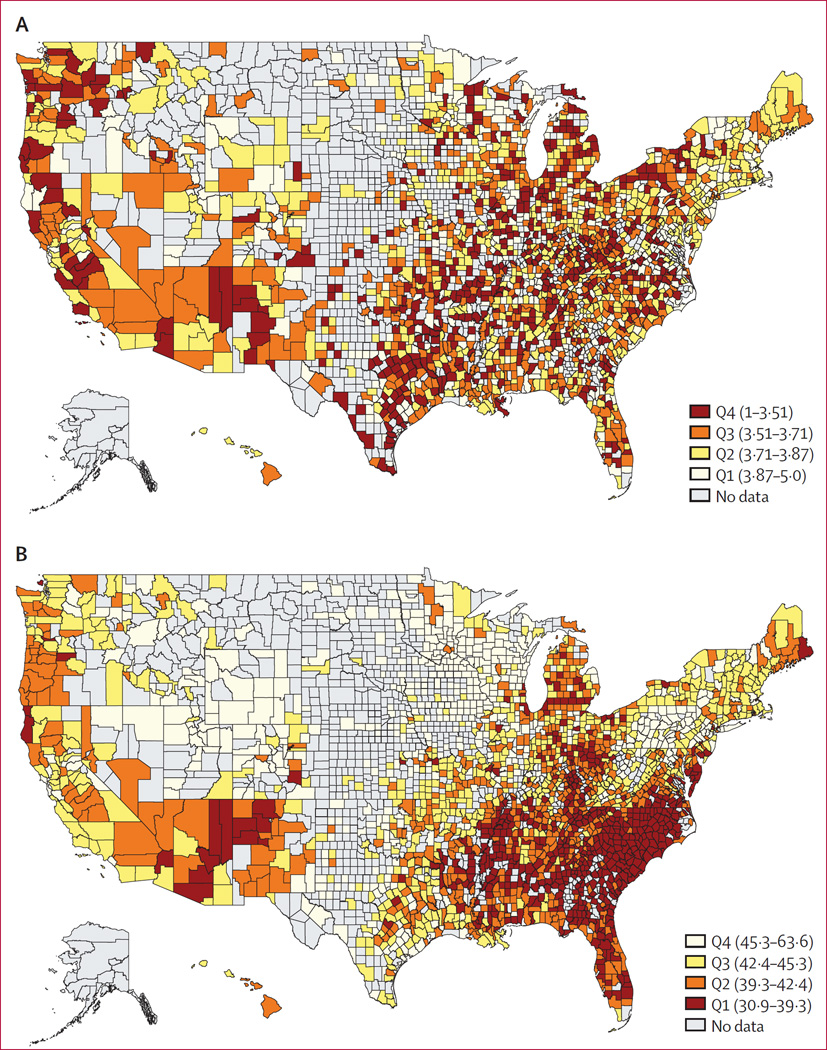
Spatial distribution of self-reported health and economic opportunity (A) Average self-reported health from the 2009–12 United States Behavioral Risk Factor Surveillance Survey across counties. (B) Average upward mobility for the 25th percentile of the income distribution. Both indicators were scaled such that red reflects poorer average health and opportunity, respectively.

**Table 1:** County-level and individual-level characterstics

	Mean (SD)	Observed number of individuals (i) or counties (c)
Economic opportunity (absolute upward rank mobility at the county level)	41·2 (3·89)	2242 c

Health outcomes		
Self-reported health (1–5 scale)	2·72 (0·98)	145 070 i
Physical health (days)	2·54 (6·25)	145 070 i
Mental health (days)	3·99 (7·88)	145 012 i

Behaviours/risk factors		
Ever smoking (%)	41·3%	145 247 i
Body-mass index (mg/kg^2^)	27·34 (5·73)	137 493 i
HIV transmission risk behaviours (%)	5·7% (0·23)	138 251 i

Individual demographics		
Age (years)	30·34 (3·07)	146 272 i
Female (%)	50%	146 272 i
Race/ethnicity		
Non-Hispanic white (%)	69%	146 272 i
Black (%)	11%	
Hispanic (%)	13%	
Other (%)	7%	146 272 i
Married (%)	54%	146 272 i

Individual socioeconomic status		
High school degree (%)	88%	146 272 i
College degree (%)	35%	146 272 i
Household income (US$)	48 212 (29 071)	146 272 i
Employed (%)	71%	146 272 i

County characteristics		
2012 Gini coefficient	0·44 (0·03)	2242 c
2010 unemployment (%)	9·85 (2·74)	2242 c
2010 log gross domestic product per head	10·18 (0·20)	2199 c

Rural–urban classification (% of counties)		
Metropolitan	45%	
Urban population >20 000	14%	2242 c
Urban population 2500–20 000	35%	2242 c
Rural	6%	2242 c

Population aged >65 years in 2005 (%)	13·92 (3·40)	2242 c

Population aged 0–14 years in 2005 (%)	19·33 (2·57)	2242 c

African-American population in 2005 (%)	10·01 (14·15)	2242 c

Log population density	4·41 (1·33)	2241 c

Social capital index	–0·37 (1·17)	2236 c

Violent crimes (per 100 000)	147·60 (126·17)	2240 c

Income segregation index	0·06 (0·04)	2241 c

Racial segregation index	0·17 (0·10)	2241 c

Primary care physician (per 100 000)	64·24 (42·42)	2242 c

Variable definitions are defined in the appendix (pp 2, 3). Health outcomes, behaviours/risk factors, individual demographics, and individual socioeconomic status all come from United States Behavioral Risk Factor Surveillance Survey individual-level data.^14^ Throughout, percentages and sample means were computed with the use of United States Behavioral Risk Factor Surveillance Survey sampling weights.

**Table 2:** Unadjusted and adjusted associations between economic opportunity and self-reported health outcomes

	Unadjusted model estimates	Demographic- adjusted model estimates*	Socioeconomic status-adjusted model estimates†
Self-reported overall health (n=146 272)	0·0070 (−0·011 to 0·003)	0·0085 (0·003 to 0·014)	0·0042 (−0·009 to 0·0003)
p value	<0·001	0·002	0·070
Change associated with interdecile increase in opportunity	0·10	0·12	0·06
Change as percentage of mean (%)	3·7%	4·4%	2·25%

Physical health days (n=145 383)	−0·015 (−0·030 to 0·001)	−0·037 (−0·069 to −0·006)	−0·024 (−0·052 to 0·004)
p value	0·060	0·020	0·098
Change associated with interdecile increase in opportunity	−0·22	−0·53	−0·35
Change as percentage of mean (%)	−8·56%	−19·6%	−13·7%

Mental health days (n=145 343)	−0·049 (−0·067 to −0·031)	−0·053 (−0·088 to −0·018)	−0·034 (−0·068 to −0·001)
p value	<0·001	0·0033	0·045
Change associated with interdecile increase in opportunity	−0·71	−0·76	−0·49
Change as percentage of mean (%)	−17·8%	−19·0%	−12·4%

Covariates			
Individual-level characteristics (not including socioeconomic status)	No	Yes	Yes
Survey year and month fixed effects	No	Yes	Yes
County-level characteristics	No	Yes	Yes
State fixed effects	No	Yes	Yes
Individual-level socioeconomic status	No	No	Yes

Data are n (95% CI), unless otherwise specified.

* Models were adjusted for individual demographics (age, sex, race, marital status), survey year and month fixed effects, county characteristics, and state fixed effects.

† Models were adjusted for individual demographics (age, sex, race, marital status), survey year and month fixed effects, county characteristics, and state fixed effects and additionally for individual socioeconomic status characteristics (household income, binary indicators for high school completion, college completion, employment, and health insurance). All models were estimated using ordinary least squares. The change in the outcome associated with an increase in the opportunity measure from the 10th to the 90th percentile is 14·5, and the scaled association size relative to the mean of the dependent variable is presented for each estimate. The full set of socioeconomic status-adjusted model estimates are presented in the appendix.

**Table 3:** Unadjusted and adjusted associations between economic opportunity and self-reported health behaviours and risk factors

	Unadjusted model estimates	Demographic- adjusted model estimates*	Socioeconomic status-adjusted model estimates†
Ever smoking (n=145 584)	−0·0019 (−0·004 to 0·0001)	−0·0044 (−0·0067 to −0·002)	−0·0026 (−0·0049 to −0·0003)
p value	0·072	<0·001	0·026
Change associated with interdecile increase in opportunity (points)	−2·8%	−5·7%	−3·8%
Change as percentage of mean (%)	−6·7%	−13·9%	−9·2%

Body-mass index (kg/m^2^; n=146 617)	−0·058 (−0·079 to −0·036)	−0·020 (−0·048 to 0·007)	−0·011 (−0·038 to 0·016)
p value	<0·001	0·14	0·42
Change associated with interdecile increase in opportunity (points)	−0·84	−0·28	−0·16
Change as percentage of mean (%)	−3%	−1%	−0·57%

HIV risk behaviours (n=138 582)	−0·0017 (−0·0023 to −0·0012)	−0·0017 (−0·0029 to −0·0006)	−0·0014 (−0·0024 to −0·0003)
p value	<0·001	0·003	0·009
Change associated with interdecile increase in opportunity (points)	−2·5%	−2·5%	−2%
Change as percentage of mean (%)	−41%	−41%	−33%

Covariates			
Individual-level characteristics (not Including socioeconomic status)	No	Yes	Yes
Survey year and month fixed effects	No	Yes	Yes
County-level characteristics	No	Yes	Yes
State fixed effects	No	Yes	Yes
Individual-level socioeconomic status	No	No	Yes

Data are n (95% CI), unless otherwise specified.

* Models were adjusted for individual demographics (age, sex, race, marital status), survey year and month fixed effects, county characteristics, and state fixed effects.

† Models were adjusted for individual demographics (age, sex, race, marital status), survey year and month fixed effects, county characteristics, and state fixed effects and additionally for individual socioeconomic status characteristics (household income, binary indicators for high school completion, college completion, employment, and health insurance). Models for ever smoking and HIV risk behaviours were estimated with a probit link function. The estimate presented is the marginal effect of the coefficient on the absolute upward mobility measure, assessed at the mean of all covariates. The change in the outcome associated with an increase in the opportunity measure from the 10th to the 90th percentile is 14·5, and the scaled association size relative to the mean of the dependent variable is presented for each estimate. The full set socioeconomic status-adjusted model estimates are presented in the appendix.

## Research in context

### Evidence before this study

We searched PubMed, Google Scholar, and EconLit with the terms “economic opportunity” OR “inequality of opportunity” AND “health” OR “mortality” OR “behaviors” OR “smoking” OR “risk factors” for all articles published before July 1, 2016. Although we found many studies focusing on the association between area-level income inequality and health, only two articles examined the association between economic opportunity and health. Both of these studies, done in the USA, showed positive associations between economic opportunity and health. One was our county-level study focused on mortality. By virtue of using aggregate data, this study was potentially subject to bias resulting from the ecological fallacy. The second was a study focused on the association between individual perceptions of economic opportunity and self-reported health in a small sample of Asian Americans living in the USA, and its generalisability to the broader US population was unclear.

### Added value of this study

We used a large, nationally representative survey of nearly 147 000 adults aged 25–35 years living in the USA, along with computed county-level estimates of intergenerational mobility, a common measure of economic opportunity. We estimated the association between economic opportunity and different measures of mental health, physical health, health behaviours, and risk factors, adjusting for a richer set of individual-level and area-level covariates than in previous work. We found that higher area-level economic opportunity was associated with improved self-reported overall health, physical health, and mental health, and reduced likelihood of smoking and undertaking risky sexual and drug use practices. Our findings represent an important contribution to the literature, in that they are nationally representative for the USA and avoid potential bias from the ecological fallacy.

### Implications of all the available evidence

Our findings illustrate potential importance of economic opportunity as an independent driver of health disparities in the USA. The results motivate future work around the role of inequality of opportunity in explaining long-standing population health disparities and recent trends in mortality among white, middle-aged US adults. Such investigations might show important and actionable linkages between policies seeking to expand economic opportunities and population health objectives.

## References

[1] Kristof N (2014). The American Dream is leaving America. http://www.nytimes.com/2014/10/26/opinion/sunday/nicholas-kristof-the-american-dream-is-leaving-america.html?_r=0.

[2] Roubini N (2016). The political left and right are being upended by globalization politics. http://www.huffingtonpost.com/nouriel-roubini/globalization-politics_b_11655494.html.

[3] Chetty R, Hendren N, Kline P, Saez E (2014). Where is the land of opportunity? The geography of intergenerational mobility in the United States. Q J Econ.

[4] Putnam R (2015). Our Kids: The American Dream in Crisis.

[5] Grossman M (1972). On the concept of health capital and the demand for health. J Polit Econ.

[6] Snyder CR, Irving LM, Anderson JR, Snyder CR, Forsyth DR (1991). Hope and health. Handbook of social and clinical psychology: the health perspective.

[7] Marmot M, Wilkinson RG (2006). Social determinants of health.

[8] Venkataramani AS, Chatterjee P, Kawachi I, Tsai AC (2016). Economic opportunity, health behaviors, and mortality in the United States. Am J Public Health.

[9] de Castro AB, Gee GC, Takeuchi DT (2010). Examining alternative measures of social disadvantage among Asian Americans: the relevance of economic opportunity, subjective social status, and financial strain for health. J Immigr Minor Health.

[10] Franzini L, Fernandez-Esquer ME (2004). Socioeconomic, cultural, and personal influences on health outcomes in low income Mexican-origin individuals in Texas. Soc Sci Med.

[11] Chetty R, Hendren N, Kline P, Saez E, Turner N (2014). Is the United States still a land of opportunity? Recent trends in intergenerational mobility. Am Econ Rev.

[12] Chetty R, Hendren N (2015). The impacts of neighborhoods on intergenerational mobility: childhood exposure effects and county-level estimates.

[13] Chetty R, Hendren N, Kline P, Saez E, Turner N The equality of opportunity project 2014. http://www.equality-of-opportunity.org/index.php/data.

[14] Centers for Disease Control and Prevention (CDC) (2009–12). Behavioral risk factor surveillance system survey data.

[15] Centers for Disease Control and Prevention (CDC) Behavioral risk factor surveillance system: 2012 summary data quality report. https://www.cdc.gov/brfss/annual_data/2012/pdf/summarydataqualityreport2012_20130712.pdf.

[16] Groves R (2006). Nonresponse rates and nonresponse bias in household surveys. Public Opin Q.

[17] US Centers for Disease Control and Prevention (2000). Measuring healthy days: population assessment of health-related quality of life.

[18] Kawachi I, Subramanian S, Berkman L, Kawachi I, Glymour M (2014). Income inequality. Social epidemiology.

[19] Marmot M, Smith G, Stansfield S (1991). Health inequalities among British civil servants: the Whitehall II study. Lancet.

[20] Wilkinson RG, Pickett K (2009). The spirit level: why more equal societies almost always do better.

[21] United States Census Bureau 2009–2013 American community survey 5-year estimates, table b19083, Gini Index of Income Inequality. http://factfinder2.census.gov.

[22] United States Bureau of Economic Analysis Local area personal income and employment.

[23] Inter-university Consortium for Political and Social Research (2008). County characteristics, 2000–2007.

[24] Inter-university Consortium for Political and Social Research (2000). Uniform crime reporting program data: county-level detailed arrest and offense data, 2000.

[25] Rupasingha A, Goetz SJ (2008). US county-level social capital data, 1990–2005.

[26] Department of Health and Human Services, Centers for Disease Control and Prevention (2010). Community health status indicators, community health data initiative. http://wwwn.cdc.gov/communityhealth.

[27] United States Census Bureau 2010 census. http://www.census.gov/2010census/.

[28] Shrier I, Platt RW (2008). Reducing bias through directed acyclic graphs. BMC Med Res Methodol.

[29] Jensen R (2012). Do labor market opportunities aff ect young women’s work and family decisions? Experimental evidence from India. Q J Econ.

[30] Sankoh A, Huque M, Dubey S (1997). Some comments on frequently used endpoint adjustment methods in clinical trials. Stat Med.

[31] Kling JR (2007). Methodologic frontiers of public finance field experiments. Natl Tax J.

[32] McLaren L (2007). Socioeconomic status and obesity. Epidemiol Rev.

[33] Schmeiser MD (2009). Expanding wallets and waistlines: the impact of family income on the BMI of women and men eligible for the earned income tax credit. Health Econ.

[34] Lynch JW, Smith GD, Kaplan GA, House JS (2000). Income inequality and mortality: importance to health of individual income, psychosocial environment, or material conditions. BMJ.

[35] Case A, Deaton A (2015). Rising morbidity and mortality in midlife among white non-Hispanic Americans in the 21st century. Proc Natl Acad Sci USA.

[36] Marrero GA, Rodriguez JG (2012). Inequality of opportunity in Europe. Rev Income Wealth.

[37] Corak M (2013). Income inequality, equality of opportunity, and intergenerational mobility. J Econ Perspect.

[38] Ward MD, Gledistch KS (2008). Spatial Regression Models.

[39] O’Brien R, Robertson C (2015). Medicaid and intergenerational economic mobility.

