# Supplementary material for: Economic opportunity, health behaviours, and health outcomes in the USA: a population-based cross-sectional study
Source: Lancet Public Health. Author manuscript; Available in PMC 2018 May 11. (PMC5947845; doi:10.1016/S2468-2667(16)30005-6)
Supplement: Supplemental Appendix [file NIHMS844011-supplement-Supplemental_Appendix.pdf]

THE LANCET Public Health

Supplementary appendix

This appendix formed part of the original submission and has been peer reviewed. We post it as supplied by the authors.

Supplement to: Venkataramani AS, Brigell R, O'Brien R, Chatterjee P, Kawachi I, Alexander C Tsai. Economic opportunity, health behaviours, and health outcomes in the USA: a population-based cross-sectional study. *Lancet Public Health* 2016; published online Oct 3. http://dx.doi.org/10.1016/S2468-2667(16)30005-6.

Supplementary Appendix

"Inequality of Economic Opportunity, Health Behaviors, and Health Outcomes in the United States: Population-Based, Cross-Sectional Study"

Authors and Affiliations:
Atheendar S. Venkataramani[1, 2], Rachel Brigell[3], Rourke O'Brien[4], Paula Chatterjee[5], Ichiro Kawachi[2,3], Alexander C. Tsai[2,6]

[1]Division of General Internal Medicine, Massachusetts General Hospital, [2]Harvard Center for Population and Development Studies, [3]Harvard T.H. Chan School of Public Health, [4]La Follette School of Public Affairs, University of Wisconsin-Madison, [5]Department of Medicine, Brigham and Women's Hospital, [6]Department of Psychiatry, Massachusetts General Hospital

Corresponding author:
Atheendar S. Venkataramani
Division of General Internal Medicine
Massachusetts General Hospital
50 Staniford St, 954-1
Boston, MA 02114
E-mail: avenkataramani@partners.org

Table A1 – Variable Descriptions and Sources

Variable Name	Description	Source
**Health Outcomes**		
Self Reported health	5-point Likert scale denoting "poor". "very poor", "good", "very good", and "excellent"	Centers for Disease Control and Prevention (CDC). Behavioral Risk Factor Surveillance System Survey Data, 2009-2013.
Physical Health Days	BRFSS variable "physhlth": Now thinking about your physical health, which includes physical illness and injury, for how many days during the past 30 days was your physical health not good?	Centers for Disease Control and Prevention (CDC). Behavioral Risk Factor Surveillance System Survey Data, 2009-2013.
Mental Health Days	BRFSS variable "menthlth": Now thinking about your mental health, which includes stress, depression, and problems with emotions, for how many days during the past 30 days was your mental health not good?	Centers for Disease Control and Prevention (CDC). Behavioral Risk Factor Surveillance System Survey Data, 2009-2013.
**Behaviors/Risk Factors**		
Ever Smoker	Variable = 1 if the individual reports ever smoking.	Centers for Disease Control and Prevention (CDC). Behavioral Risk Factor Surveillance System Survey Data, 2009-2013.
BMI	Kg/m2. Calculated using self-reported height and weight.	Centers for Disease Control and Prevention (CDC). Behavioral Risk Factor Surveillance System Survey Data, 2009-2013.
HIV Risk	Variable = 1 if the respondent answered yes to the following questions: I am going to read you a list. When I am done, please tell me if any of the situations apply to you. You do not need to tell me which one. You have used intravenous drugs in the past year. You have been treated for a sexually transmitted or venereal disease in the past year. You have given or received money or drugs in exchange for sex in the past year. You had anal sex without a condom in the past year. Do any of these situations apply to you?"	Centers for Disease Control and Prevention (CDC). Behavioral Risk Factor Surveillance System Survey Data, 2009-2013.
**Opportunity**		
Absolute Upward Mobility	The county-average expected national income rank of children born to parents in the lowest quartile of the income distribution; higher values reflect greater economic opportunity. Computed using 2010-2012 income tax data for 1980-1982 birth cohorts along with linked tax returns of their parents.	Chetty, et al (2014)

Rank-Rank Slope	The county-average correlation in household income ranks between parents and their children. Computed using 2010-2012 income tax data for 1980-1982 birth cohorts along with linked tax returns of their parents.	Chetty, et al (2014)
% Change in Income, Causal Movers	The percent change in household income owing to moving to county X before the age of 13 from reference county Y. Computed using sibling comparisons among families making cross-county moves. Computed using 2010-2012 income tax data for 1980-1982 birth cohorts along with linked tax returns of their parents.	Chetty, et al (2015)

County-Level Characteristics

County Per Capita Income	For 2010	Bureau of Economic Analysis
Gini Coefficient 2010	For 2010	American Community Survey, 2009-2012, 5 yr estimates
% Unemployed	For 2010	Bureau of Economic Analysis
Urbanization	For year 2003; 4 variables created from ERS classification as follows: 1 = Counties located in metropolitan areas; 2 = Nonmetropolitan counties with urban pop of >20K, 3 = Nonmetropolitan counties with urban pop of 2,500-20,000K; 4 = Rural, less than 2,500 urban pop	ICPSR 20660; County Characteristics, 2000-2007
% Over 65 Years of Age	For year 2005	ICPSR 20660; County Characteristics, 2000-2007
% Under 15 Years of Age	For year 2005	ICPSR 20660; County Characteristics, 2000-2007
% African American	For year 2005	ICPSR 20660; County Characteristics, 2000-2007
Population Density	For year 2000, persons per square mile	ICPSR 20660; County Characteristics, 2000-2007
Social Capital Index	Standardized index (normalized to mean 0, s.d. 1) combining measures of voter turnout rates, the fraction of people who return their census forms, and measures of participation in community organizations. See Rupasingha and Goetz. For year 1990	Penn State NRCRD; Rupasingha and Goetz (2008)
Violent Crimes	Total number of violent crimes (murder, rape, robbery, aggravated assault) per 100,000 in 2000	ICPSR 3451; Uniform Crime Reporting Program Data
Income Segregation	For year 2000, at commuter zone level. Rank order index at census tract level as in Reardon (2011)	Chetty, et al (2014)
Racial Segregation	For year 2000, at commuter zone level. Theil index at census tract level for 4 races (White, Black, Hispanic, Other)	Chetty, et al (2014)
Primary Care Physicians per 100,000	For year 2007	CDC and HHS Comm Health Status Indicators 2009

Notes:
Weblinks for Data Sources:
CDC Compressed Mortality File: http://wonder.cdc.gov/mortsql.html

CDC and HHS Comm Health Status Indicators 2009: http://wwwn.cdc.gov/CommunityHealth/homepage.aspx?j=1
Chetty, et al (2014 and 2015): Papers and public use data set at www.equality-of-opportunity.org
US Census Bureau, ACS 5-year estimates: www2.census.gov/programs-surveys/acs/summary_file/2013/data/
Bureau of Economic Analysis: http://www.bea.gov/iTable/index_nipa.cfm.
ICPSR 20660, County Characteristics, 2000-2007: http://www.icpsr.umich.edu/icpsrweb/ICPSR/studies/20660
USDA Economic Research Service Education Data: http://www.ers.usda.gov/data-products.aspx
ICPSR 3451, Uniform Crime Reporting Program Data: http://www.icpsr.umich.edu/icpsrweb/ICPSR/series/57/studies/3451
Penn State NRCRD and Rupasingha and Goetz Social Capital Data: http://aese.psu.edu/nercrd/economic-development/materials/poverty-issues/big-boxes/wal-mart-and-social-capital/social-capital/social-capital-variables-spreadsheet/view

Table A2 – Sample Sizes and Missing Data

Sample Restriction	Observations	% of Initial
Initial Sample	187,143	
County Identifier Present	164,857	88.1%
Opportunity Measure Present	164,789	88.1%
Individual-Level Income Measure Present	151,434	80.9%
County Gini, Income Per Capita, Social Capital, Health Present	147,613	78.9%
All Restrictions	146,272	78.2%

Notes:
This table demonstrates the loss of BRFSS observations for self-reported health with the use of individual and county-level covariates. The first line lists the full number of available observations. The second lists the number (first column) and percentage (of total; second column) of available observations with a non-missing county identifier. 12% of sample respondents are lost at this step because the BRFSS, for privacy considerations, does not identify some small counties. As noted in the main text, the counties we do identify comprise of 95% of the U.S. population. The next greatest loss of observations comes from the use of individual-level data on household income (91% of all BRFSS observations). With all covariates, our final sample consists of 78% of all available observations for the self-reported health measure.

Table A3 – Robustness of Results to Missing Observations

	Self-Rep Health	Physical Health Days	Mental Health Days	Smoking	BMI	HIV Risk
**All Observations**						
Absolute Upward Mobility	0.006	-0.021	-0.05	-0.004	-0.026	-0.0011
95% CI	(0.003, 0.009)	(-0.037, -0.006)	(-0.069, -0.032)	(-0.006, -0.0028)	(-0.048, -0.004)	(-0.0016, -0.007)
P-value	[<0.001]	[0.007]	[<0.001]	[<0.001]	[0.023]	[<0.001]
Observations	163,480	162,277	162,205	162,611	152,925	154,441
**Estimation Sample**						
Absolute Upward Mobility	0.0057	-0.018	-0.035	-0.004	-0.026	-0.0011
95% CI	(0.002, 0.009)	(-0.034, -0.001)	(-0.054, -0.017)	(-0.006, -0.0025)	(-0.049, -0.002)	(-0.0016, -0.0053)
P-value	[0.001]	[0.035]	[<0.001]	[<0.001]	[0.030]	[<0.001]
Observations	146,272	145,070	145,012	145,247	137,493	138,251

Notes:
The models in this table regress each of the outcomes in the header on economic opportunity and individual-level age, gender, race, and marital status for all 25-35 year olds in the BRFSS. Each column-panel represents a separate regression. The top panel ("All Observations") uses all available data. The bottom panel ("Estimation Sample") uses the same observations (for which we have full data on all covariates) as in the main text. Note that the sample size for self-reported health is similar to the figure of 164,789 noted in **Table S2** (the difference is due to the small percentage of non-response for the aforementioned demographic controls). The main point of this table is to show that the point estimates on the outcomes of interest are substantively similar regardless of the sample used. This argues against the possibility of missing data driving the findings.

Table A4 – Full Regression Results

	Self-Reported Health	Physical Health Days	Mental Health Days	Ever Smoking	BMI	HIV Risk
Absolute Upward Mobility	0·0042	-0·024*	-0·034*	-0·026**	-0·011	-0·0014**
	(-0·0003, 0·0087)	(-0·052, 0·0044)	(-0·068, -0·0008)	(-0·0049, -0·00032)	(-0·038, 0·016)	(-0·0024, -0·0034)
**Individual-Level Characteristics**						
Male Gender	-0·0048	-0·23***	-0·83**	0·11**	0·84**	0·010**
	(- 0·022, 0·013)	(-0·35, -0·098)	(-0·97, -0·70)	(0·095, 0·12)	(0·72, 0·95)	(0·0061, 0·015)
Race: Black	-0·020	-0·72**	-0·76**	-0·22**	1·81**	0·013**
	(-0·051, 0·012)	(-0·93, -0·50)	(-1·04, -0·48)	(-0·24, -0·21)	(1·61, 2·01)	(0·0073, 0·019)
Race: Hispanic	-0·15**	-0·57**	-1·53**	-0·21**	1·00**	0·00020
	(-0·19, -0·11)	(-0·81, -0·34)	(-1·82, -1·25)	(-0·23, -0·19)	(0·77, 1·23)	(-0·0063, 0·0067)
Race: Other	-0·10**	-0·062	-0·50**	-0·077**	-0·55**	-0·0056
	(-0·014, -0·070)	(-0·30, 0·18)	(-0·74, -0·27)	(-0·096, -0·059)	(-0·77, -0·33)	(-0·014, 0·0029)
Married	0·10**	-0·43**	-1·29**	-0·095**	-0·024	-0·035**
	(0·083, 0·12)	(-0·57, -0·30)	(-1·45, -1·12)	(-0·11, 0·085)	(-0·13, 0·084)	(-0·040, -0·029)
HH Income < $25,000	[REF]	[REF]	[REF]	[REF]	[REF]	[REF]
HH Income $25,000-$49,999	0·26**	-1·14**	-1·36**	-0·028**	-0·34**	-0·0042
	(0·23, 0·29)	(-1·34, -0·95)	(-1·60, -1·13)	(-0·041, -0·016)	(-0·53, -0·16)	(-0·0095, 0·0011)
HH Income $50,000+	0·44**	-1·62**	-1·97**	-0·063**	-0·71**	-0·0041

	(0·41, 0·47)	(-1·81, -1·43)	(-2·23, -1·71)	(-0·077, -0·048)	(-0·91, -0·52)	(-0·010, 0·0020)
Employed	0·14**<br>(0·12, 0·16)	-1·58**<br>(-1·75, -1·40)	-1·55**<br>(-1·77, -1·33)	-0·024**<br>(-0·036, -0·011)	0·023<br>(-0·11, 0·16)	-0·0079**<br>(-0·013, -0·0033)
< High School Education	[REF]	[REF]	[REF]	[REF]	[REF]	[REF]
High School Education	0·27**<br>(-0·31, -0·24)	-0·85**<br>(-1·15, -0·56)	-0·78**<br>(-1·11, -0·46)	-0·067**<br>(-0·087, -0·047)	0·16<br>(-0·055, 0·37)	-0·015**<br>(-0·023, -0·0066)
College Education	0·21**<br>(0·20, 0·23)	-0·56**<br>(-0·67, -0·46)	-0·90**<br>(-1·04, -0·77)	-0·22**<br>(-0·24, -0·21)	-1·23**<br>(-1·35, -1·12)	-0·016**<br>(-0·020, -0·012)
Health Insurance	-0·043**<br>(-0·064, -0·022)	-0·20<br>(-0·45, 0·043)	0·15<br>(-0·075, 0·37)	0·027**<br>(0·017, 0·037)	-0·31**<br>(-0·42, -0·19)	0·0053**<br>(0·0017, 0·0089)
**County-Level Characteristics**						
Gini (2012)	0·64**<br>(0·21, 1·08)	-3·60**<br>(-5·98, -1·22)	-2·47<br>(-6·04, 1·11)	-0·39**<br>(-0·62, -0·17)	-1·26<br>(-3·78, 1·25)	-0·043<br>(-0·14, 0·056)
Unemployment (2010)	-0·0029<br>(-0·0049, 0·011)	0·0084<br>(-0·038, 0·055)	-0·013<br>(-0·078, 0·051)	0·0043*<br>(0·00066, 0·0079)	0·019<br>(-0·025, 0·063)	-0·00049<br>(-0·0019, 0·00097)
Log Income (2010)	0·0048<br>(-0·08, 0·090)	0·61*<br>(0·12, 1·09)	0·13<br>(-0·48, 0·74)	0·031<br>(-0·014, 0·075)	-0·88**<br>(-1·37, -0·40)	0·0046<br>(-0·013, 0·022)
Metropolitan County	[REF}	[REF}	[REF}	[REF}	[REF}	[REF}

Urban Population >20,000	-0·0089<br>(-0·042, 0·024)	-0·12<br>(-0·34, 0·090)	-0·0072<br>(-0·29, 0·28)	0·0021<br>(-0·016, 0·020)	0·0053<br>(-0·22, 0·23)	0·0010<br>(-0·0070, 0·0091)
Urban Population 2,500-20,000	0·0049<br>(-0·032, 0·042)	-0·095<br>(-0·33, 0·14)	-0·044<br>(-0·39, 0·30)	-0·026*<br>(-0·046, -0·0059)	-0·12<br>(-0·37, 0·14)	-0·00098<br>(-0·0090, 0·0071)
Rural	0·0097<br>(-0·076, 0·095)	0·27<br>(-0·47, 1·02)	-0·72*<br>(-1·33, -0·10)	-0·047*<br>(-0·090, -0·0045)	0·17<br>(-0·43, 0·77)	-0·010<br>(-0·028, 0·0072)
Population Aged 65+ Years (%)	-0·0052*<br>(-0·011, -0·000049)	0·0030<br>(-0·027, 0·033)	0·0049<br>(-0·029, 0·039)	0·0031*<br>(0·00062, 0·0056)	0·033*<br>(0·0030, 0·063)	0·00049<br>(-0·00058, 0·0016)
Population Aged 0-14 Years (%)	0·0043<br>(-0·100, 0·005)	-0·0082<br>(-0·040, 0·024)	0·0091<br>(-0·038, 0·056)	-0·00041<br>(-0·0037, 0·0029)	0·027<br>(-0·0093, 0·063)	0·00032<br>(-0·00093, 0·0016)
African American Population (%)	0·0012*<br>(-0·000050, 0·0024)	-0·0011<br>(-0·0086, 0·0063)	-0·0079<br>(-0·017, 0·0015)	-0·00069*<br>(-0·0014, 0·0000084)	-0·0025<br>(-0·011, 0·0058)	0·000076<br>(-0·00019, 0·00034)
Log Population Density	-0·011*<br>(-0·023, 0·00084)	-0·0035<br>(-0·074, 0·068)	0·050<br>(-0·039, 0·14)	-0·0015<br>(-0·0082, 0·0052)	-0·13**<br>(-0·19, -0·066)	-0·0011<br>(-0·0037, 0·0015)
Social Capital Index	0·0040<br>(-0·012, 0·020)	-0·055<br>(-0·16, 0·045)	-0·065<br>(-0·19, 0·060)	0·0076*<br>(-0·0057, 0·016)	-0·0078<br>(-0·11, 0·099)	-0·0016<br>(-0·0053, 0·0021)
Violent Crimes (Per 100,000)	-0·000016<br>(-0·00013, 0·000094)	-0·000078<br>(-0·00059, 0·00044)	0·00017<br>(-0·00049, 0·00082)	0·000042*<br>(0·0000077, 0·000091)	-0·000070<br>(-0·000094, 0·00013)	-0·000019*<br>(-0·000038, 0·000001)

Income Segregation	0·19	-2·99	-3·65	-0·0016	-1·92	0·099
	(-0·36, 0·74)	(-6·36, 0·37)	(-8·21, 0·91)	(-0·31, 0·31)	(-5·56, 1·72)	(-0·030, 0·23)
Racial Segregation	-0·0098	0·26	0·85	0·023	0·67	-0·025
	(-0·15, 0·17)	(-0·73, 1·24)	(-0·43, 2·13)	(-0·063, 0·11)	(-0·35, 1·69)	(-0·061, 0·012)
Primary Care Physicians (Per 100,000)	0·00033*	0·00064	-0·000099	-0·00001	-0·0016	0·000082*
	(-0·000011, 0·00064)	(-0·0012, 0·0025)	(-0·0024, 0·0022)	(-0·00016, 0·00014)	(-0·0035, 0·00032)	(0·0000023, 0·00016)
Observations	146,272	145,383	145,343	145,584	146,617	138,582

Notes:
Models are identical to those estimated in Column 3 of Tables 2 and 3, except here the coefficient estimates on all covariates are shown. Each column represents a separate regression, with the dependent variables noted in the column header. The estimates ever smoking and HIV risk factors were obtained from marginal effects probit models evaluated at the mean of the independent variables. All other coefficients were computed using Ordinary Least Squares. 95% confidence intervals, computed using standard errors corrected for clustering at the county level, are in parenthesis. Given the size of the table, we denote p-values as follows: ** - p<0·01, * - p<0·05. All models additionally control for state fixed effects and survey year and month fixed effects.

Table A5 – Multiple Hypothesis Testing

	Self-Rep. Health	Physical Health Days	Mental Health Days	Smoking	BMI	HIV Risk	Index
Absolute Upward Mobility	0.0085	-0.037	-0.006	-0.007	-0.02	-0.0017	-0.015
P-value	0.002	0.02	0.003	<0.001	0.142	0.003	<0.001
Adjusted P-value	0.012	0.11	0.017	<0.001	0.585	0.017	N/A
Observations	146,209	145,007	144,950	145,184	137,438	138,195	127,983

Notes:
Models are identical to those estimated in Column 2 of Tables 2 and 3. Each column represents a separate regression.
Here we conduct two different multiple hypothesis tests. The first uses a group-wise Bonferroni method following Sankoh, Huque, and Dubey (1997, *Statistics in Medicine 16*(22)). This method adjusts p-values in the following manner:

$$P\ adjusted = 1 - (1 - p(k))^{M^{1-r(.k)}}$$

where *p(k)* is the (unadjusted) p-value for outcome *k*; *M* is the number of outcomes being tested; and *r(.k)* is the mean correlation among all outcomes other than *k.* Note that when *r(.k)* = 0, this method reverts back to a simple Bonferroni correction. The adjusted p-values are presented below the unadjusted values. With the exception of physical health days, the adjusted values remain statistically significant by convention thresholds ($p<0.05$).

The second method we use follows Kling (2007, *National Tax Journal* 60(1)). The basic idea is to create a single latent index from a family of outcomes. Statistical significance of the latent index would support the contention that findings for any individual outcomes are not due to chance. We use the first principal component of all six outcomes as our latent index. The results of a regression of this index on the opportunity measure and covariates listed in Col. 2 of Tables 2 and 3 in the main text are shown in the final column. We found a substantively and statistically significant association between our exposure measure and this index (the negative sign owes to the fact that physical and mental health days, smoking, BMI, and HIV risk are all negative outcomes; the self-reported health measure follows suit and loads negatively in the first principal component). Of note, the grouping of variables to create indices is subjective. For example, one could group self-reported health, physical health days, and BMI to create a “physical health” index and the mental health, smoking, and HIV risk variables to create a “mental health and substance use” index. Doing so results in statistically significant associations for both indices. However, we opt to go with the single index as it is less arbitrary in its construction.

Table A6– Estimates Using Alternate Measures of Economic Opportunity

	Self-Reported Health	Physical Health Days	Mental Health Days	Smoking	BMI	HIV Risk
**MEASURE**						
Rank-Rank Slope	0·41	1·24	2·06	0·21	2·36	0·079
	(0·17, 0·66)	(-0·4, 2·87)	(0·22, 3·90)	(0·087, 0·33)	(0·92, 3·81)	(0·021, 0·14)
	[0·001]	[0·14]	[0·029]	[0·0007]	[0·0014]	[0·007]
Observations	146,272	145,383	145,343	145,584	146,617	138,582
% Income Change, Causal Movers	0·053	-0·22	-0·26	-0·039	-0·082	-0·008
	(0·0005, 0·10)	(-0·51, 0·074)	(-0·55, 0·034)	(-0·056, -0·021)	(-0·32, 0·15)	(-0·018, 0·002)
	[0·048]	[0·14]	[0·083]	[0·000]	[0·50]	[0·12]
Observations	146,209	145,007	144,950	145,184	137,438	138,195

Notes:
Models are identical to those estimated in Column 2 of Tables 2 and 3, except here alternate measures of economic opportunity (both derived from the same database as the absolute upward mobility measure) are used. The first is the county-level intergenerational correlation in income or income rank (“Rank-Rank Slope”, obtained from Chetty, et al (2014), which reflects relative mobility. Higher values of this measure reflect lower opportunity for those in the bottom of the income distribution, as it implies that children of poor parents were more likely to stay poor. (Conversely, higher values also reflect greater opportunity for those in the top of the income distribution, as it implies that children of richer parents were more likely to become rich.) The second is Chetty and Hendren’s (2015) measure of the expected change in adult income attributable to growing up in a particular county % Change in Income, Causal Movers). This measure was computed comparing adult incomes across siblings in families who moved from one county to another during their childhood. Higher values reflect greater mobility.

As in Tables 2 and 3, each column x panel represents a separate regression, with the dependent variables noted in the column header. The estimates for Smoking, and HIV Risk were obtained from marginal effects probit models. These coefficients (multiplied by 100) convey the percentage point change in the probability of the outcome as a result of a 1 unit change in the exposure variable. The coefficients for the remaining outcomes were computed using Ordinary Least Squares. 95% confidence intervals, computed using standard errors corrected for clustering at the county, are parenthesis. Exact p-values are provided in square brackets.

Table A7 – Estimates Restricting Sample to 2011 and 2012 BRFSS

	Self-Reported Health	Physical Health Days	Mental Health Days	Smoking	BMI	HIV Risk
Absolute Upward Mobility	0·0093	-0·052	-0·058	-0·0046	-0·0087	-0·0015
95% CI	(0·001, 0·017)	(-0·09, -0·014)	(-0·11, -0·0061)	(-0·0075, -0·0017)	(-0·042, 0·025)	(-0·0029, 0·00001)
P-value	[0·016]	[0·008]	[0·028]	[0·002]	[0·61]	[0·057]
Observations	146,272	145,383	145,343	145,584	146,617	138,582

Notes:
Models are identical to those estimated in Column 2 of Tables 2 and 3 of the main paper, except here we restricted the sample to the 2011-2012 BRFSS only. Starting in 2011, the BRFSS expanded their sample frame to include cell-phone users. These results thus indicate the robustness of our findings to differences in sample frame. The estimates for Smoking, and HIV Risk were obtained from marginal effects probit models. These coefficients (multiplied by 100) convey the percentage point change in the probability of the outcome as a result of a one unit change in the exposure variable. The coefficients for the remaining outcomes were computed using Ordinary Least Squares. 95% confidence intervals, computed using standard errors corrected for clustering at the county, are parenthesis. Exact p-values are provided in square brackets.

Table A8 – Association Between Economic Opportunity and Migration

	Cross-County Migration within last year = 1	Migration from Birth State = 1
**American Community Survey (ACS)**		
**Absolute Upward Mobility**	-0·00037	0·00225
	(-0·0019, 0·0012)	(-0·0035, 0·0084)
	[0·64]	[0·45]
Observations	1,360,339	1,360,339
**Current Population Survey (CPS)**		
Absolute Upward Mobility	0·001	
	(0·0025, 0·0047)	
	[0·54]	
*Absolute Upward Mobility*Self Reported Health*	-0·00046	
	[-0·0012, 0·0003]	
	[0·24]	
Self Reported Health	0·0083	
	[-0·001, 0·018]	
	[0·081]	
Observations	82,764	

Notes:
To examine the association between economic opportunity and migration, which may bias our findings if healthy individuals tend to sort into higher opportunity areas, we utilized data from the 2009-2014 waves of American Community Survey (ACS, https://usa.ipums.org/usa/) and the 2009-2015 waves of Current Population Survey (CPS, https://cps.ipums.org/). Both surveys ask respondents whether they moved within the last year and, if so, whether they moved across counties. We used this information to create a binary indicator (=1) denoting in-migration within the last year. Unfortunately, information on cross-county migration is unavailable. For the ACS, we additionally constructed an indicator of cross-state migration since the time of birth, creating a binary indicator denoting presently living in a state different than the birth state. Regardless, these data do not allow us to assess cross-county migration period to one year before enumeration or identify cross-state moves leading back to the birth state. We qualify our findings on those grounds.
We estimated probit models with the same covariates as in Column 2 of Table 2. Panel A presents results from the ACS, and we find no associations between county opportunity and migration; if anything the point estimate is negative. The CPS surveys respondents on self-reported health, the same Likert Scale measure used in our main analyses (Tables 2 and 3). We thus assessed whether the association between in-migration in the last year and economic opportunity varied by self-reported health (Panel B). A caveat here is that health status is measured at the time of survey, and therefore after the move. However, if moves have a causal positive effect on health, or if health shocks induced certain times of moves and then regressed to the mean, our coefficient estimates would be biased away from the null. As seen in the second panel, while we find a positive association between better self-reported health and in-migration, this association does not vary by county-economic opportunity.
All models include the same covariates as Table 2, Column 2. 95% CIs, which are in parenthesis, are corrected for clustering at the county level. Exact p-values are provided in square brackets.

Table A9 – Regional Variation in Association Between Economic Opportunity and Health

	Self-Reported Health	Physical Health Days	Mental Health Days	Smoking	BMI	HIV Risk
**Full Sample**						
Absolute Upward Mobility	0.0075	-0.035	-0.066	-0.004	-0.019	-0.0015
95% CI	(0.0025, 0.012)	(-0.066, -0.003)	(-0.10, -0.029)	(-0.006, -0.001)	(-0.046, 0.008)	(-0.0026, -0.0004)
P-value	[0.003]	[0.031]	[0.000]	[0.001]	[0.16]	[0.01]
Absol. Up. Mobil * South	0.0020	-0.0044	0.00099	-0.00020	-0.0061	-0.00041
95% CI	(-0.007, 0.011)	(-0.049, 0.040)	(-0.050, 0.05)	(-0.004, 0.003)	(-0.064, 0.051)	(-0.002, 0.001)
P-value	[0.65]	[0.85]	[0.97]	[0.92]	[0.83]	[0.60]
Observations	146,272	145,383	145,343	145,584	146,617	138,582
**South Only**						
Absolute Upward Mobility	0.0087	-0.055	-0.089	-0.0049	-0.024	-0.0017
95% CI	(-0.033, 0.021)	(-0.11, 0.017)	(-0.16, -0.02)	(-0.01, -0.0002)	(-0.086, 0.039)	(-0.004, 0.0002)
P-value	[0.16]	[0.057]	[0.012]	[0.041]	[0.45]	[0.085]
Observations	50,663	50,212	50,220	50,476	47,267	48,103

Notes:
Each column-panel represents a separate regression, with the dependent variable provided in the header. Models are identical to those presented in Col 2 of Tables 2 and 3 in the main text, except here we (1) additionally interact our core economic opportunity measure with a binary indicator for residence in the US South census region and (2) estimate the core models for individuals in the US South only. The motivation for these models is the fact that economic opportunity tends to be lower in the US South than in other parts of the country (see Figure 1, and these models allow us to assess whether our results are being driven by this region or whether the relationship between economic opportunity and health differs here.

In the first panel, the main effect for the South dummy is subsumed by state FE. We see that the interaction between the opportunity measure and the binary indicator for residence in the US South is small and statistically insignificant throughout. In the second panel, we see that the coefficients on economic opportunity are relatively similar in magnitude to those estimated in column 2 of Tables 2 and 3, though, owing to smaller sample sizes, less precisely estimated. The results collectively demonstrate that our national findings are not driven by the U.S. South.

Table A10 - Estimates for Individuals Ages 65-75 Years

	Self-Reported Health	Physical Health Days	Mental Health Days	Smoking	BMI	HIV Risk	Index
**Older Adults**							
Absolute Upward Mobility	0.004	-0.016	-0.006	-0.007	0.009	0.0001	-0.001
95% CI	(-0.001, 0.009)	(-0.056, 0.025)	(-0.030, 0.018)	(-0.010, -0.005)	(-0.014, 0.033)	(-0.0002, 0.0004)	(-0.007, 0.004)
P-value	[0.09]	[0.46]	[0.61]	[<0.001]	[0.44]	[0.65]	[0.63]
Observations	309,517	302,501	305,191	308,382	304, 994	146,228	289,366

Notes:
Models are identical to those estimated in Column 2 of Tables 2 and 3 of the main paper, except here we estimated regressions using all individuals 65-75 years of age. The idea is to examine the relationship between economic opportunity and health among a group of individuals for whom opportunity may be less salient – a placebo test. Individuals 65 and above were chosen given that this is a dominant age of retirement in the United States and, consequently, the bulk of their careers are behind them. Consequently, we would expect a reduced relationship between economic opportunity and each of the health outcomes. The results generally conform to this expectation. We do find a large, negative and statistically significant association between opportunity and smoking. This could be due to random chance or the fact that inequality of opportunity may persist over long-periods of time (see Chetty et al, 2014, *American Economic Review* 104(5)) and the addictive nature of smoking lends itself to state dependence. Supporting the possibility of random chance is the fact that a summary index of all outcomes (see Table S5 notes) yields a coefficient on the economic opportunity measure, which is no different than zero.

As a note of caution to this exercise, it may be that mortality selection yields a sample that is positively selected on health, thus for whom economic opportunity (and other social determinants of health) may be less important. However, we note that a large literature does find persistent socioeconomic gradients in health even at these ages (Banks et al, 2006, *JAMA* 295(17)).
